# Supplementary material for: Single cell multiomic analysis reveals diabetes-associated β-cell heterogeneity driven by HNF1A
Source: Nat Commun. 2023 Sep 5;14:5400. doi: 10.1038/s41467-023-41228-3 (PMC10480445; doi:10.1038/s41467-023-41228-3)
Supplement: Supplementary file 1 — Supplementary Information [file 41467_2023_41228_MOESM1_ESM.pdf]

Supplementary Information

Single cell multiomic analysis reveals diabetes-associated β-cell heterogeneity driven by *HNF1A*

Supplementary Fig. 1 scRNA-seq and snATAC-seq filtering strategies and quality control.

Supplementary Fig. 2. Peak-Gene pair analysis.

Supplementary Fig. 3. 3D genome analysis in α- and β-cells.

Supplementary Fig. 4. A network of T2D upregulated peak-gene pairs.

Supplementary Fig. 5. Role of HNF1A in Type II Diabetes.

Supplementary Fig. 6. HNF1A Cut&Run in EndoC-βH3.

Supplementary Fig. 7. Additional Patch-seq data analysis related to *HNF1A* functions.

Supplementary Table 1. eHi-C reads summary.

Supplementary Table 2. Tn5 oligos.

Supplementary Table 3. PCR oligos.

Supplementary Table 4. qPCR primers.

Supplementary Table 5. Antibodies used in this study.

Supplementary Table 6. Patient Donor Information used in this study.

Supplementary Fig 1

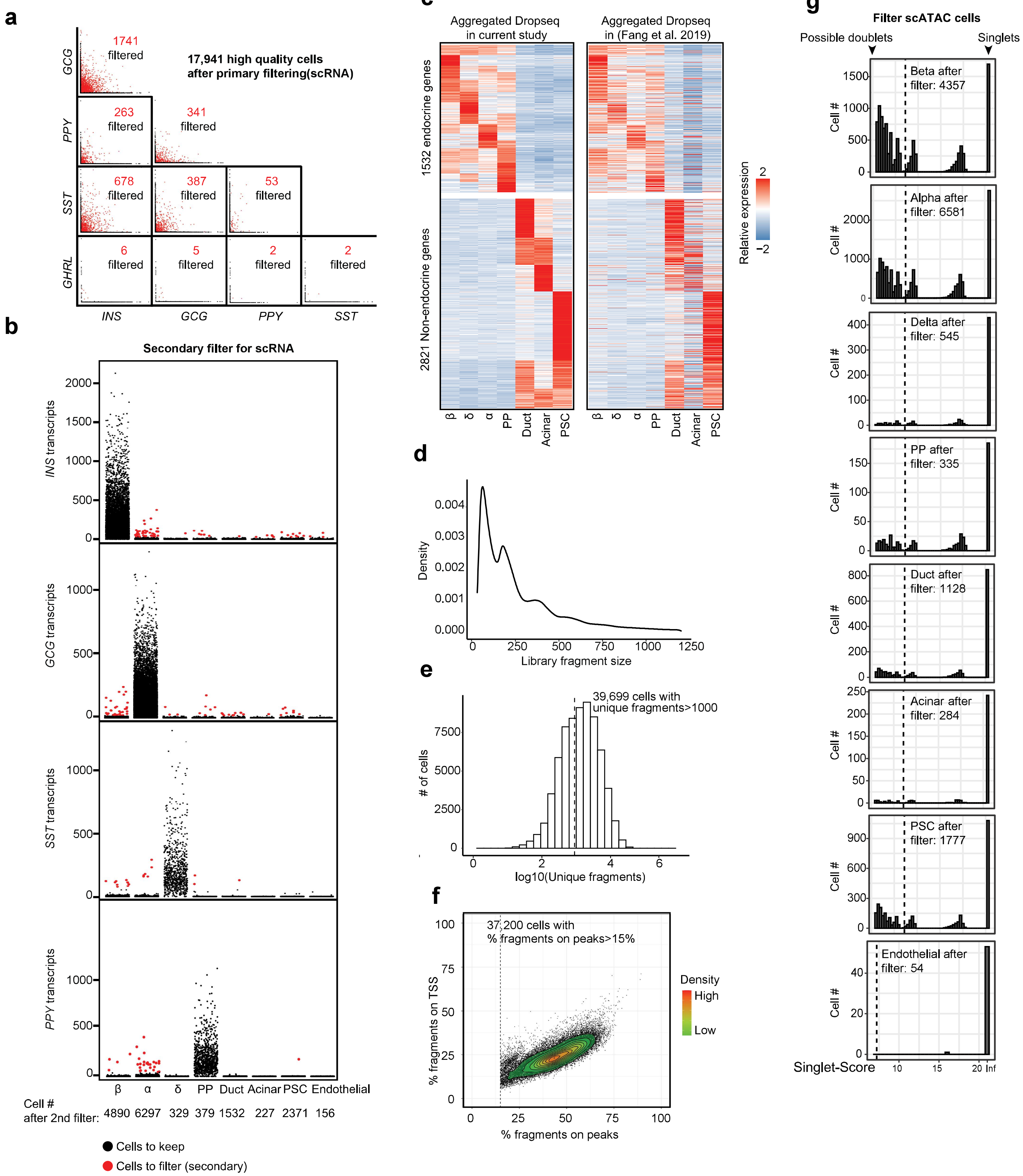

Supplementary Fig. 1 scRNA-seq and snATAC-seq filtering strategies and quality control. (**a**) Hormone-based doublets filtering strategy for scRNA-seq data. In principle, any cell that expresses two or more hormone gene is labeled as a doublet and filtered. (**b**) Secondary doublets filtering for scRNA-seq data. After clustering and cell-type annotation using the global transcriptome landscapes, if a classified cell is highly expressing a hormone gene not consistent with the defined cell type, this cell is filtered. (**c**) Cell type specific gene expression profile comparison between current cohort and our previously published cohort. Aggregated RPKM is used for visualization and comparison. (**d-f**) snATAC-seq quality control. (**d**) snATAC data fragment size distribution. (**e**) Distribution of the number of unique fragments per cell. (**f**) snATAC-seq data filtering using the percentage on peaks versus the percentage on TSS. (**g**) snATAC-seq doublets filtering based on the KNN distance ratio (**Methods**).

Supplementary Fig 2

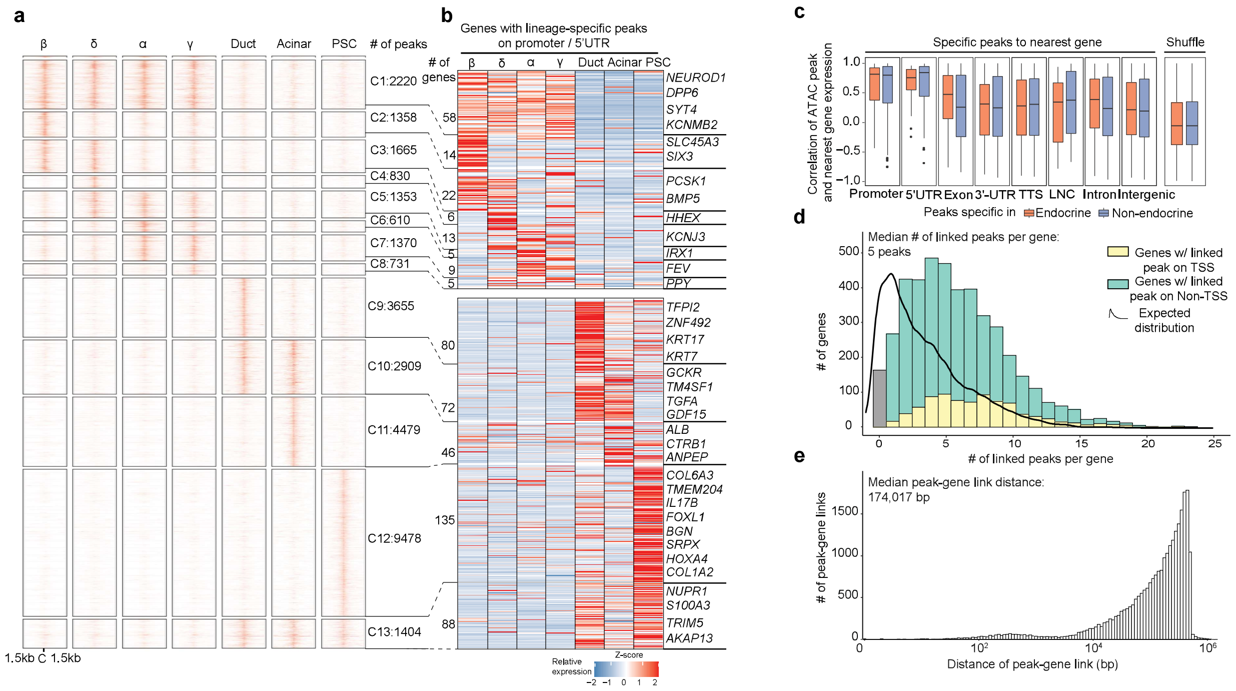

Supplementary Fig. 2. Peak-Gene pair analysis.

(**a-b**) Identify thirteen categories of cell type specific peaks (**a**) and their corresponding genes (**b**) if the peaks are located at promoter (-1kb to +100bp) or 5' UTRs. (**c**) Correlation (Pearson's r) between cell-type specific ATAC peaks and their nearest genes stratified by the peak locations. (**d-e**) Peak-gene link analysis based on correlation instead of proximity (**Methods**). (**d**) Summary of the number of linked peaks for each gene. (**e**) Summary of the genomic distance between the peak-gene pairs.

Supplementary Fig 3

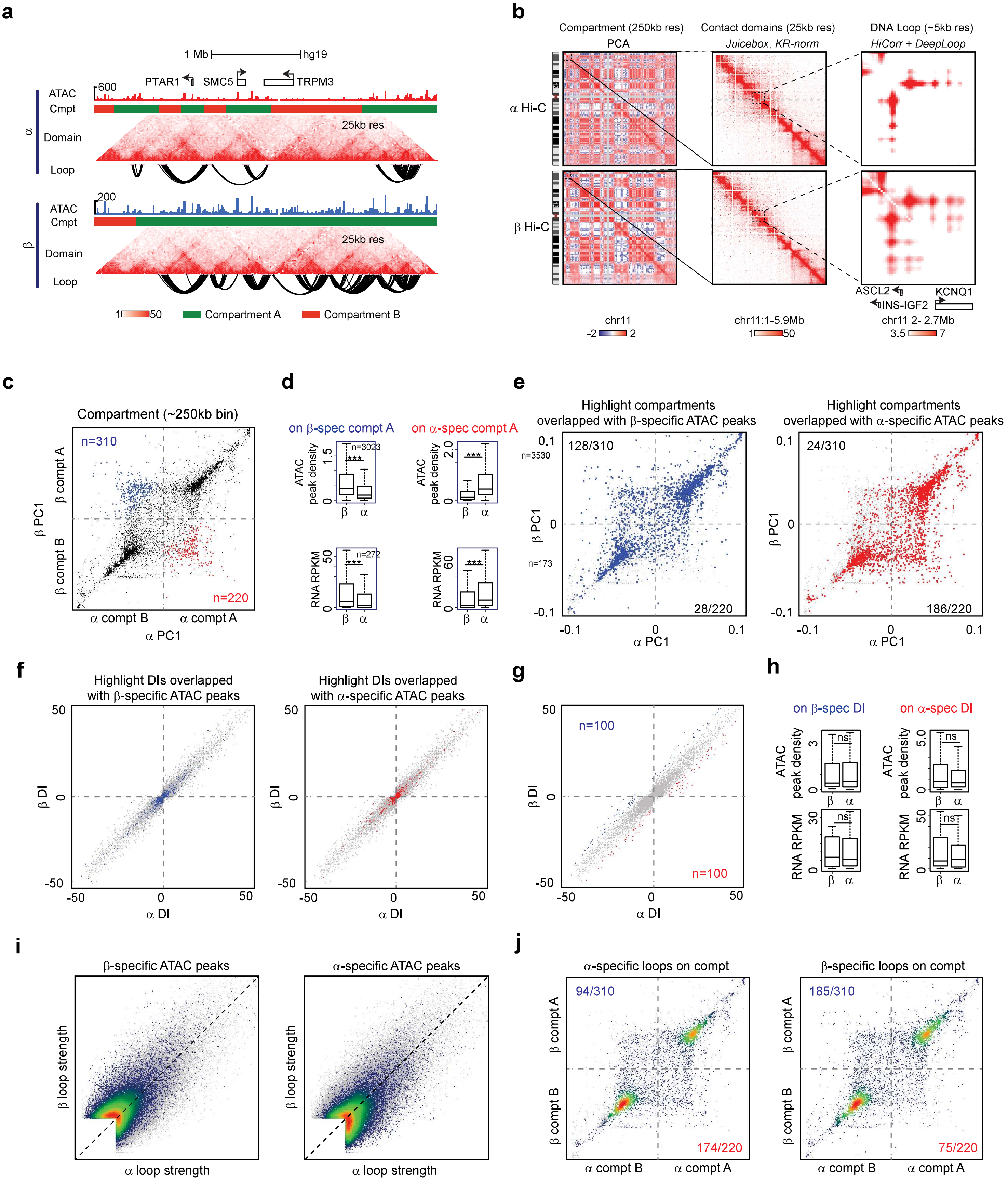

Supplementary Fig. 3. 3D genome analysis in α- and β-cells.

(**a**) Genomic tracks showing ATAC, compartment A/B, contact heatmaps, and chromatin loop curves in α-cell and β-cell at locus chr9:73129495-74491882. (**b**) Hierarchical genome architecture (compartment, contact domains and DNA Loops) near *INS-IGF2* locus in both α- and β-cells. (**c**) Scatterplot comparing PC1 values from compartment analyses in α- and β-cells. Highlighted dots are the α-cell or β-cell specific compartment A bins (250kb resolution). (**d**) Boxplots comparing the ATAC intensity (top two panels) and gene expression (bottom two panels) with the 220 α-cell or β-cell specific compartment A regions from (**c**). (**e**) The scatterplots compare the PC1 values of α-cell and β-cell Hi-C data in compartment-level analyses (each dot represents a 250kb bin). Blue highlights bins overlapping 5,000 β-cell specific ATAC peaks. Red highlights bins are overlapping 5,000 α-cell specific ATAC peaks. (**f**) The scatterplots compare the DI (direction index) values of α-cell and β-cell Hi-C data in TAD-level analysis (each dot represents a 25kb bin). Left panel highlights the bins overlapping β-cell specific ATAC peaks; Right panel highlights the bins overlapping α-cell specific ATAC peaks. (**g**) Define a small number of bins with α-cell specific (red) and β-cell specific (blue) DI values. (**h**) Compare the ATAC peak strength and gene expression within the bins with α-cell or β-cell specific DI values. (**i**) Scatterplot comparing the strength of loops between α-cells and β-cells. Heat colored dots are loops overlapping 5,000 β-cell specific ATAC peaks (left panel) or 5,000 α-cell specific ATAC peaks (right panel). (**j**) Scatter plots highlighting the α-cell or β-cell specific loops from **Fig. 3b** to examine their correlation with compartment switching events.

Supplementary Fig 4

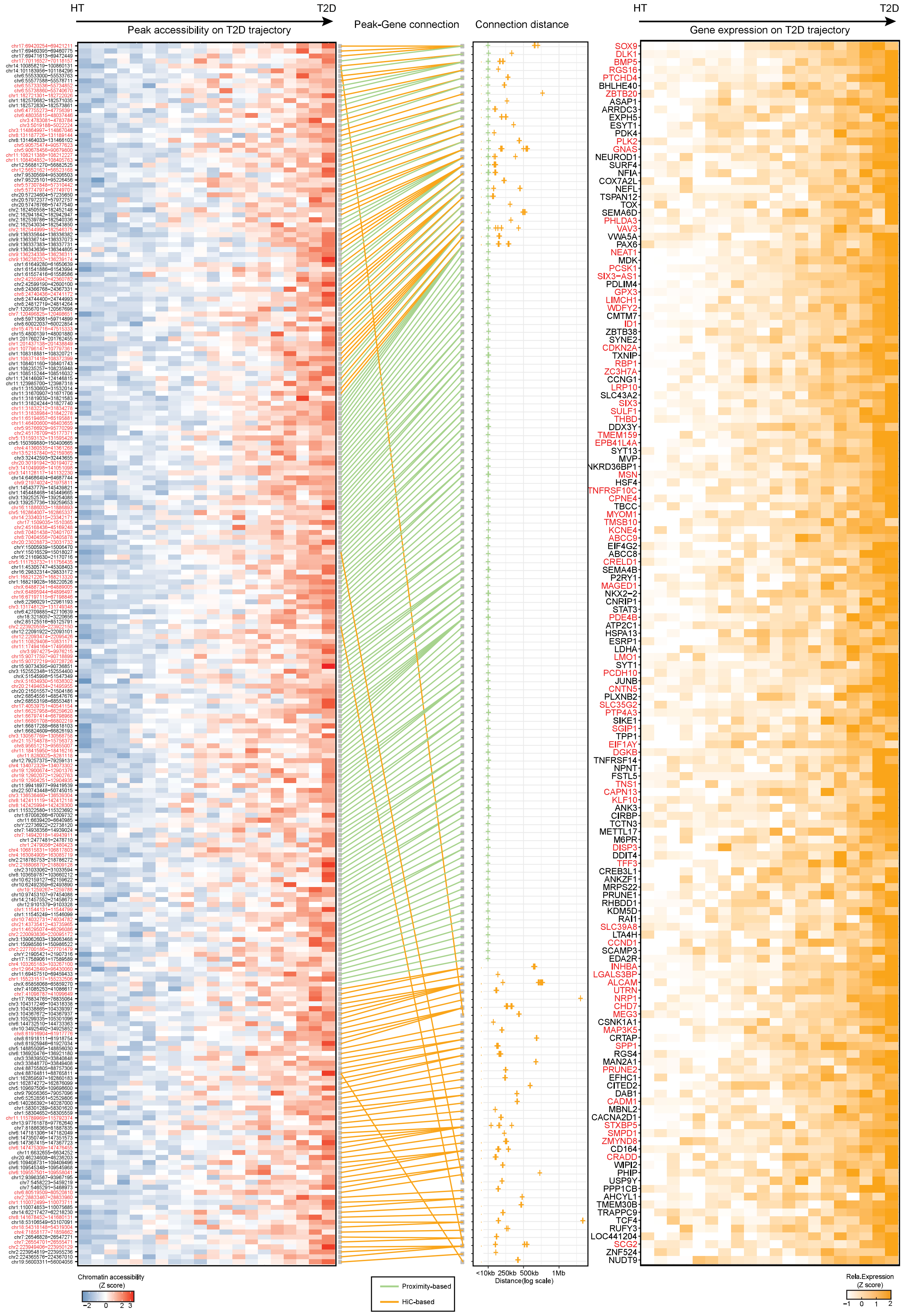

Supplementary Fig. 4. A network of T2D upregulated peak-gene pairs.

Connect T2D up-regulated ATAC peaks to up-regulated genes from RePACT analyses through multiomic integration (similar as **Fig. 6a**). Leftmost panel: heatmap of peaks gaining accessibility along T2D trajectory; peak locations (hg19) are on the left of each row. Peaks highlighted in red show intra-donor heterogeneity. Second panel shows the peak-gene connections. Green lines: peaks within 10kb of the TSS; orange lines: distal peak-gene pairs supported by Hi-C loops. Third panel: the distance of each peak-TSS connection. Rightmost panel: heatmap of T2D up-regulated genes. Red-highlighted gene names indicate intra-donor heterogeneous genes.

Supplementary Fig 5

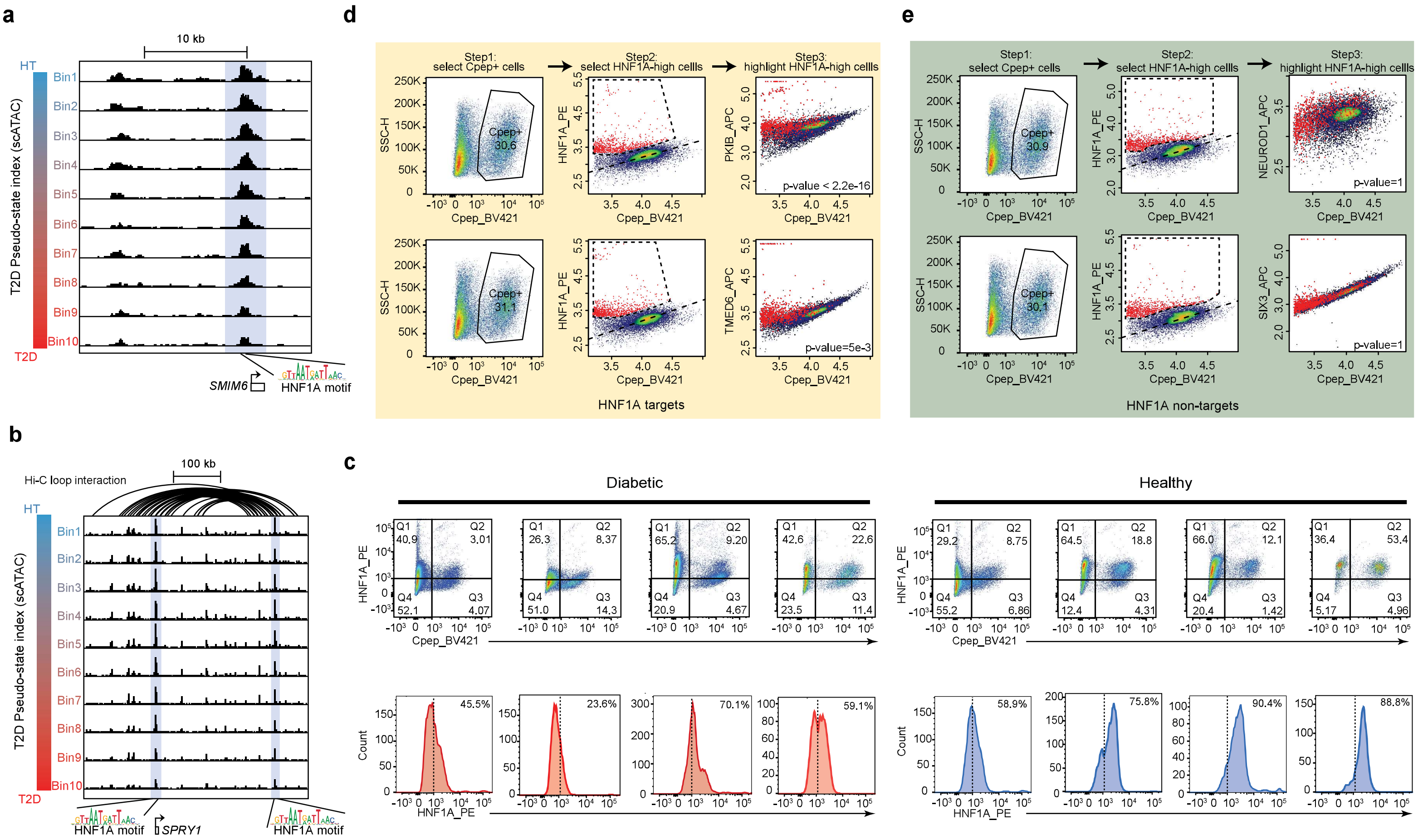

Supplementary Fig. 5. Role of HNF1A in Type II Diabetes

Similar as **Fig. 6d**, genome browser snapshots of putative *HNF1A* target genes *SMIM6* (**a**) and *SPRY1* (**b**). (**c**) Flow cytometry scatterplot (top row) of HNF1A and C-pep islet cells from 4 healthy and 4 diabetic donors. The histogram (bottom row) shows the distribution of HNF1A signal in the β-cells (gated with C-peptide) from 4 healthy (blue) and 4 diabetic (red) donors. (**d-e**) Similar as **Fig. 7c-d**, additional β-cell three-way flow cytometry results for (**d**) *HNF1A* target genes PKIB and TMED6 in non-diabetic islets, and (**e**) control genes NEUROD1 and SIX3 in non-diabetic islets. P values from one sided *t.test*.

Supplementary Figure 6

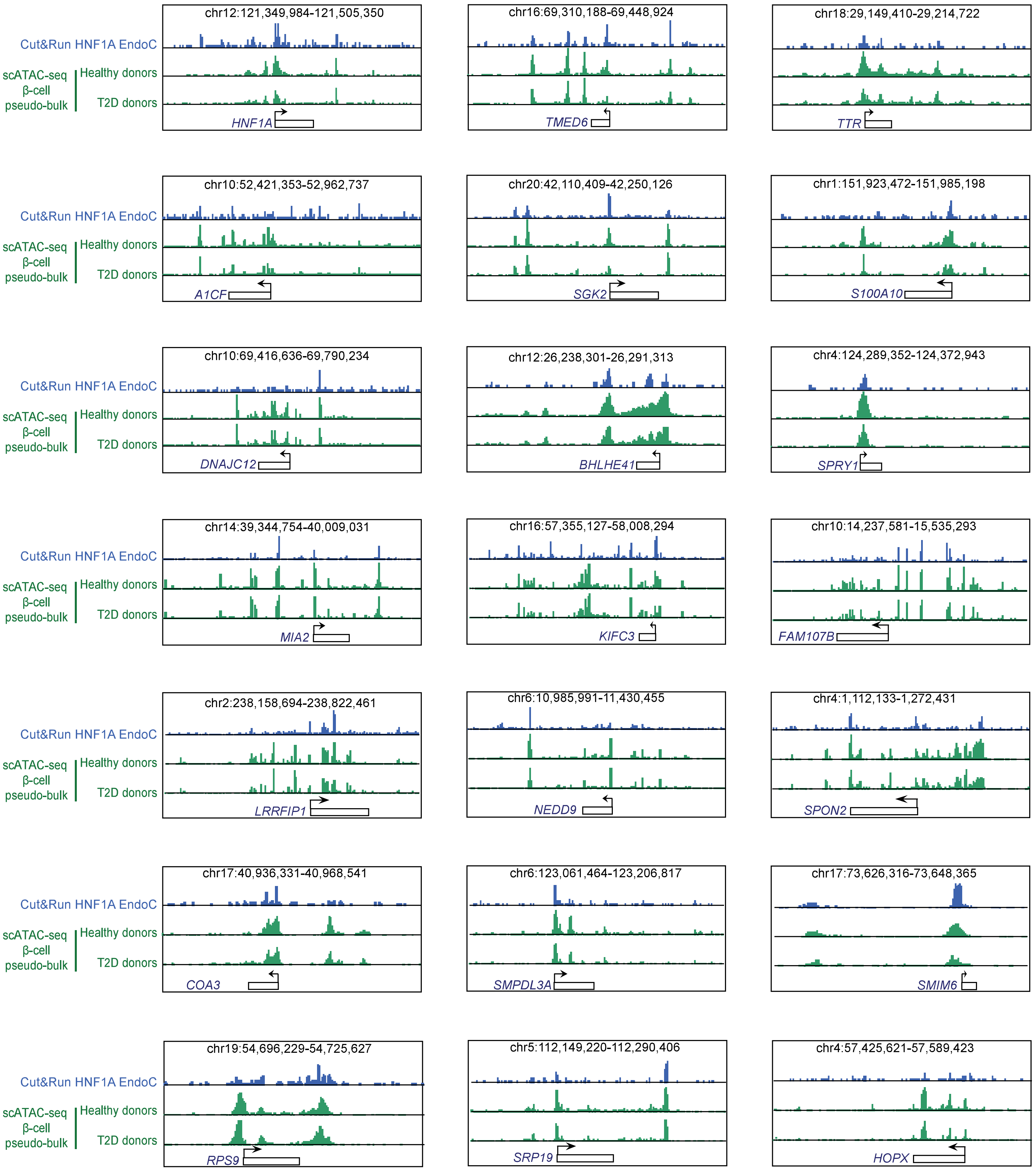

Supplementary Fig. 6. HNF1A Cut&Run in EndoC-βH3

Genome browser snapshots of select *HNF1A* target gene loci. Each track has HNF1A Cut&Run (blue) and pseudo-bulk ATAC tracks from T2D and healthy β cells (green).

Supplementary Fig 7

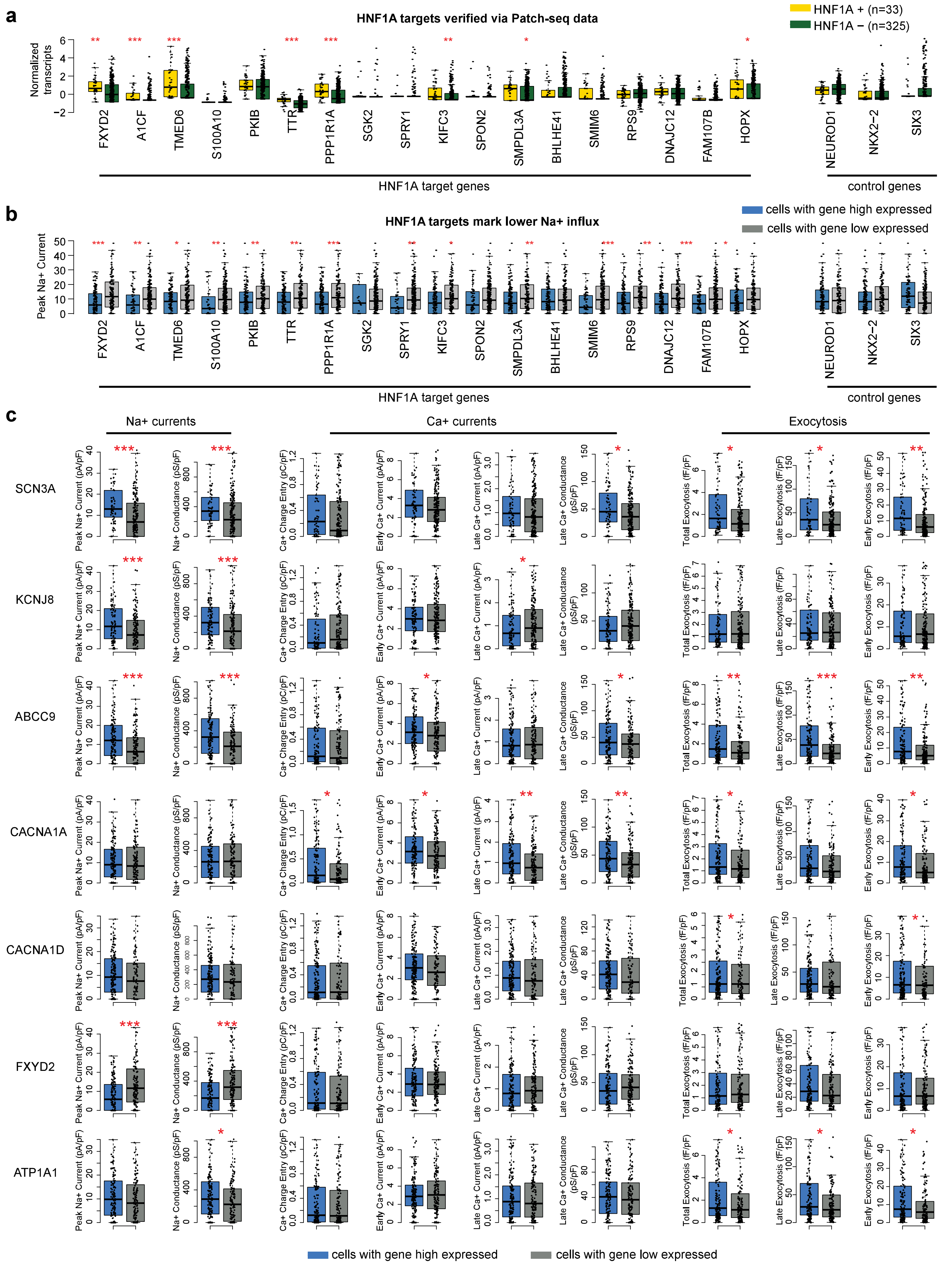

Supplementary Fig. 7. Additional Patch-seq data analysis related to *HNF1A* functions.

(**a**) Expression of *HNF1A* target genes and three non-target control genes in *HNF1A*+ β cells (yellow, n=33) and *HNF1A*- β cells (green, n=325) from Patch-seq data (**Methods**). ***p < 0.001, **p < 0.01, *p < 0.05 (Mann-Whitney U test). (**b**) Boxplots showing the association between *HNF1A* target genes and Na+ current measurement from Patch-seq data. For each gene of interest, β-cells are divided into high expression group (blue) and low expression group (grey) (**Methods**). ***p < 0.001, **p < 0.01, *p < 0.05 (Mann-Whitney U test). (**c**) For every denoted channel gene, cells are divided into high- and low-expression groups (**Methods**). The boxplots show if the gene expression is associated with a variety of electrophysiological measurements from Patch-seq data. ***p < 0.001, **p < 0.01, *p < 0.05 (Mann-Whitney U test).

Supplementary Table 1. eHi-C reads summary

Cell type	Sample	Total reads	Uniquely mapped	Non-redundant	Cis	Trans	Cis-2Mb
Alpha	Hi-C.alpha1	405,505,134	211,513,369	165,434,689	265,909,112	129,064,344	153,872,881
	Hi-C.alpha2	350,990,532	170,669,624	148,809,750			
	Hi-C.alpha3	398,703,410	72,194,498	60,303,123			
	Hi-C.alpha4	368,176,579	219,650,622	187,385,773			
	Hi-C.alpha5	417,280,536	272,466,837	231,279,903			
	Hi-C.alpha6	437,437,508	229,231,601	192,318,080			
	Hi-C.alpha7	430,008,729	194,518,534	172,247,820			
Beta	Hi-C.beta1	397,634,633	184,709,869	147,274,958	195,737,809	87,367,515	120,099,961
	Hi-C.beta2	385,862,924	57,501,715	50,498,651			
	Hi-C.beta3	387,875,392	131,038,273	105,789,167			
	Hi-C.beta4	404,610,467	254,126,546	210,718,872			
	Hi-C.beta5	461,766,459	253,441,320	215,582,251			
	Hi-C.beta6	476,561,356	219,768,827	173,865,774			

Supplementary Table 2. Tn5 oligos

Oligo name	Sequence
pMENTS	5Phos/CTGTCTCTTATACACATCT
Tn5_p7_1	GTCTCGTGGGCTCGG**AAACAC**GTCTCCGCCTCAGATGTGTATAAGAGACAG
Tn5_p7_2	GTCTCGTGGGCTCGG**AAAGGG**GTCTCCGCCTCAGATGTGTATAAGAGACAG
Tn5_p7_3	GTCTCGTGGGCTCGG**AACCGA**GTCTCCGCCTCAGATGTGTATAAGAGACAG
Tn5_p7_4	GTCTCGTGGGCTCGG**CCAATA**GTCTCCGCCTCAGATGTGTATAAGAGACAG
Tn5_p7_5	GTCTCGTGGGCTCGG**CCTCTT**GTCTCCGCCTCAGATGTGTATAAGAGACAG
Tn5_p7_6	GTCTCGTGGGCTCGG**CGTGAT**GTCTCCGCCTCAGATGTGTATAAGAGACAG
Tn5_p7_7	GTCTCGTGGGCTCGG**GCTGAA**GTCTCCGCCTCAGATGTGTATAAGAGACAG
Tn5_p7_8	GTCTCGTGGGCTCGG**GGACAA**GTCTCCGCCTCAGATGTGTATAAGAGACAG
Tn5_p7_9	GTCTCGTGGGCTCGG**GGCATT**GTCTCCGCCTCAGATGTGTATAAGAGACAG
Tn5_p7_10	GTCTCGTGGGCTCGG**TGTCTA**GTCTCCGCCTCAGATGTGTATAAGAGACAG
Tn5_p7_11	GTCTCGTGGGCTCGG**TTACCC**GTCTCCGCCTCAGATGTGTATAAGAGACAG
Tn5_p7_12	GTCTCGTGGGCTCGG**TTTCAG**GTCTCCGCCTCAGATGTGTATAAGAGACAG
Tn5_p5_a	TCGTCGGCAGCGTC**AAGATC**TCGAGGACGGCAGATGTGTATAAGAGACAG
Tn5_p5_b	TCGTCGGCAGCGTC**AATTCC**TCGAGGACGGCAGATGTGTATAAGAGACAG
Tn5_p5_c	TCGTCGGCAGCGTC**CTAAGC**TCGAGGACGGCAGATGTGTATAAGAGACAG
Tn5_p5_d	TCGTCGGCAGCGTC**CTTGTG**TCGAGGACGGCAGATGTGTATAAGAGACAG
Tn5_p5_e	TCGTCGGCAGCGTC**GAATAG**TCGAGGACGGCAGATGTGTATAAGAGACAG
Tn5_p5_f	TCGTCGGCAGCGTC**GATGCT**TCGAGGACGGCAGATGTGTATAAGAGACAG
Tn5_p5_g	TCGTCGGCAGCGTC**TCCAAG**TCGAGGACGGCAGATGTGTATAAGAGACAG
Tn5_p5_h	TCGTCGGCAGCGTC**TGGGTT**TCGAGGACGGCAGATGTGTATAAGAGACAG

Supplementary Table 3. PCR oligos

Name	Sequence
Nextera_N701	CAAGCAGAAGACGGCATACGAGAT**TCGCCTTA**GTCTCGTGGGCTCGG
Nextera_N702	CAAGCAGAAGACGGCATACGAGAT**CTAGTACG**GTCTCGTGGGCTCGG
Nextera_N703	CAAGCAGAAGACGGCATACGAGAT**TTCTGCCT**GTCTCGTGGGCTCGG
Nextera_N704	CAAGCAGAAGACGGCATACGAGAT**GCTCAGGA**GTCTCGTGGGCTCGG
Nextera_N705	CAAGCAGAAGACGGCATACGAGAT**AGGAGTCC**GTCTCGTGGGCTCGG
Nextera_N706	CAAGCAGAAGACGGCATACGAGAT**CATGCCTA**GTCTCGTGGGCTCGG
Nextera_N707	CAAGCAGAAGACGGCATACGAGAT**GTAGAGAG**GTCTCGTGGGCTCGG
Nextera_N708	CAAGCAGAAGACGGCATACGAGAT**CCTCTCTG**GTCTCGTGGGCTCGG
Nextera_N709	CAAGCAGAAGACGGCATACGAGAT**AGCGTAGC**GTCTCGTGGGCTCGG
Nextera_N710	CAAGCAGAAGACGGCATACGAGAT**CAGCCTCG**GTCTCGTGGGCTCGG
Nextera_N711	CAAGCAGAAGACGGCATACGAGAT**TGCCTCTT**GTCTCGTGGGCTCGG
Nextera_N712	CAAGCAGAAGACGGCATACGAGAT**TCCTCTAC**GTCTCGTGGGCTCGG
Nextera_N713	CAAGCAGAAGACGGCATACGAGAT**TCATGAGC**GTCTCGTGGGCTCGG
Nextera_N714	CAAGCAGAAGACGGCATACGAGAT**CCTGAGAT**GTCTCGTGGGCTCGG
Nextera_N715	CAAGCAGAAGACGGCATACGAGAT**TAGCGAGT**GTCTCGTGGGCTCGG
Nextera_N716	CAAGCAGAAGACGGCATACGAGAT**GTAGCTCC**GTCTCGTGGGCTCGG
Nextera_N717	CAAGCAGAAGACGGCATACGAGAT**TACTACGC**GTCTCGTGGGCTCGG
Nextera_N718	CAAGCAGAAGACGGCATACGAGAT**AGGCTCCG**GTCTCGTGGGCTCGG
Nextera_N719	CAAGCAGAAGACGGCATACGAGAT**GCAGCGTA**GTCTCGTGGGCTCGG
Nextera_N720	CAAGCAGAAGACGGCATACGAGAT**CTGCGCAT**GTCTCGTGGGCTCGG
Nextera_N721	CAAGCAGAAGACGGCATACGAGAT**GAGCGCTA**GTCTCGTGGGCTCGG
Nextera_N722	CAAGCAGAAGACGGCATACGAGAT**CGCTCAGT**GTCTCGTGGGCTCGG
Nextera_N723	CAAGCAGAAGACGGCATACGAGAT**GTCTTAGG**GTCTCGTGGGCTCGG
Nextera_N724	CAAGCAGAAGACGGCATACGAGAT**ACTGATCG**GTCTCGTGGGCTCGG
Nextera_N725	CAAGCAGAAGACGGCATACGAGAT**TAGCTGCA**GTCTCGTGGGCTCGG
Extended_N726	CAAGCAGAAGACGGCATACGAGAT**CTCATTGT**GTCTCGTGGGCTCGG
Extended_N727	CAAGCAGAAGACGGCATACGAGAT**GAGTGTAG**GTCTCGTGGGCTCGG
Extended_N728	CAAGCAGAAGACGGCATACGAGAT**GCTAGGTT**GTCTCGTGGGCTCGG
Extended_N729	CAAGCAGAAGACGGCATACGAGAT**GGAAGGAT**GTCTCGTGGGCTCGG
Extended_N730	CAAGCAGAAGACGGCATACGAGAT**GGTCACTC**GTCTCGTGGGCTCGG
Extended_N731	CAAGCAGAAGACGGCATACGAGAT**GTTTCGTT**GTCTCGTGGGCTCGG
Extended_N732	CAAGCAGAAGACGGCATACGAGAT**TCAAACAT**GTCTCGTGGGCTCGG
Extended_N733	CAAGCAGAAGACGGCATACGAGAT**TCCCATGT**GTCTCGTGGGCTCGG
Extended_N734	CAAGCAGAAGACGGCATACGAGAT**TGTTTCCC**GTCTCGTGGGCTCGG
Extended_N735	CAAGCAGAAGACGGCATACGAGAT**AAACAAAC**GTCTCGTGGGCTCGG
Extended_N736	CAAGCAGAAGACGGCATACGAGAT**TTTACATC**GTCTCGTGGGCTCGG
Nextera_N501	AATGATACGGCGACCACCGAGATCTACAC**TAGATCGC**TCGTCGGCAGCGTC
Nextera_N502	AATGATACGGCGACCACCGAGATCTACAC**CTCTCTAT**TCGTCGGCAGCGTC
Nextera_N503	AATGATACGGCGACCACCGAGATCTACAC**TATCCTCT**TCGTCGGCAGCGTC
Nextera_N504	AATGATACGGCGACCACCGAGATCTACAC**AGAGTAGA**TCGTCGGCAGCGTC
Nextera_N505	AATGATACGGCGACCACCGAGATCTACAC**GTAAGGAG**TCGTCGGCAGCGTC
Nextera_N506	AATGATACGGCGACCACCGAGATCTACAC**ACTGCATA**TCGTCGGCAGCGTC
Nextera_N507	AATGATACGGCGACCACCGAGATCTACAC**AAGGAGTA**TCGTCGGCAGCGTC
Nextera_N508	AATGATACGGCGACCACCGAGATCTACAC**CTAAGCCT**TCGTCGGCAGCGTC
Nextera_N509	AATGATACGGCGACCACCGAGATCTACAC**CGTCTAAT**TCGTCGGCAGCGTC
Nextera_N510	AATGATACGGCGACCACCGAGATCTACAC**TCTCTCCG**TCGTCGGCAGCGTC
Nextera_N511	AATGATACGGCGACCACCGAGATCTACAC**TCGACTAG**TCGTCGGCAGCGTC
Nextera_N512	AATGATACGGCGACCACCGAGATCTACAC**TTCTAGCT**TCGTCGGCAGCGTC
Nextera_N513	AATGATACGGCGACCACCGAGATCTACAC**CCTAGAGT**TCGTCGGCAGCGTC
Nextera_N514	AATGATACGGCGACCACCGAGATCTACAC**GCGTAAGA**TCGTCGGCAGCGTC
Nextera_N515	AATGATACGGCGACCACCGAGATCTACAC**CTATTAAG**TCGTCGGCAGCGTC
Nextera_N516	AATGATACGGCGACCACCGAGATCTACAC**AAGGCTAT**TCGTCGGCAGCGTC

Nextera_N517	AATGATACGGCGACCACCGAGATCTACAC**GAGCCTTA**TCGTCGGCAGCGTC
Nextera_N518	AATGATACGGCGACCACCGAGATCTACAC**TTATGCGA**TCGTCGGCAGCGTC
Extended_N519	AATGATACGGCGACCACCGAGATCTACAC**TTTACATC**TCGTCGGCAGCGTC
Extended_N520	AATGATACGGCGACCACCGAGATCTACAC**AAACAAAC**TCGTCGGCAGCGTC
Extended_N521	AATGATACGGCGACCACCGAGATCTACAC**AGCAAATT**TCGTCGGCAGCGTC
Extended_N522	AATGATACGGCGACCACCGAGATCTACAC**CGTTGGTG**TCGTCGGCAGCGTC
Extended_N523	AATGATACGGCGACCACCGAGATCTACAC**GACTTCTT**TCGTCGGCAGCGTC
Extended_N524	AATGATACGGCGACCACCGAGATCTACAC**GGTCACTC**TCGTCGGCAGCGTC

Supplementary Table 4. qPCR primers

Primer	Sequence 5' to 3'
HNF1A F1	GGTCCTACGTTCACCAACACA
HNF1A R1	CTCTGGGTCACATGGCTCT
S100A10 F	GGCTACTTAACAAAGGAGGACC
S100A10 R	GAGGCCCGCAATTAGGGAAA
A1CF F	TGTGGACAACTGCCGATTATTT
A1CF R	TGACATCGACAACACCTTCAGTA
TTR F	TGGGAGCCATTTGCCTCTG
TTR R	AGCCGTGGTGGAATAGGAGTA
DNAJC12 F	AGCATCCTGAAAACCCCAAAG
DNAJC12 R	GCGAGAAACCCACCGTCTT
BHLHE41 F	AAGGAGCATGAAACGAGACGA
BHLHE41 R	CTCGGTTAAGGCGGTTAAAGC
KIF3 F	GTGGAGCTGCGACTCAAGG
KIF3 R	CTGCTTGGTCTTGGACGACT
PPP1R1A F	CCACGGCAACGGAAGAAGAT
PPP1R1A R	CCCCAGGTGATGTTCAACCA
SGK2 F	ACTCCACCCTTCAACCCAAAT
SGK2 R	GCGCATAAGAAAATCCCAGGAA
TMED6 F	GGGCTGGTCGTTCTGAATCTA
TMED6 R	CGCTGAACCTCGTAACTGAAAT
PKIB F	GAGTCTGGGGTCGCCAATTTT
PKIB R	TGAACTCTGGATGTCTGGTAAGG
GAPDH F	GGAGCGAGATCCCTCCAAAAT
GAPDH R	GGCTGTTGTCATACTTCTCATGG
ACTIN F	CATGTACGTTGCTATCCAGGC
ACTIN R	CTCCTTAATGTCACGCACGAT
FXYD2 F	ATCCTCCTCAGTAAGTGGGGT
FXYD2 R	CTTGGCAACTCCCGAAAGC

Supplementary Table 5. Antibodies used in this study

Flow Cytometry Antibodies

Target	Primary Ab	Secondary Ab
HNF1A	Cat# 89670S, CST	Cat# 406421, Biolegend
CPEP	Cat# GN-ID4-s, DSHB	Cat# 565013, BD Biosciences
TTR	Cat# sc-377517, Santa Cruz	Cat# A865, Invitrogen
SIX3	Cat# sc-398797, Santa Cruz	Cat# A865, Invitrogen
TMED6	Cat# SAB1408365, Sigma	Cat# A865, Invitrogen
PKIB	Cat# SAB1412738, Sigma	Cat# A865, Invitrogen
A1CF	Cat# SAB1400523, Sigma	Cat# A865, Invitrogen
NEUROD1	Cat# 563566, BD Biosciences	--
NKX2.2	Cat# 564729, BD Biosciences	--

Primary Ab

Name	Catalog #	Lot #	Clone #	Dilution	Manufacturer
HNF1A	Cat# 89670S	#1	NA	1:50	CST
CPEP	Cat# GN-ID4-s	NA	NA	1:50	DSHB
TTR	Cat# sc-377517	#J0316	NA	1:50	Santa Cruz
SIX3	Cat# sc-398797	#B0922	NA	1:50	Santa Cruz
TMED6	Cat# SAB1408365	#10027	NA	1:50	Sigma
PKIB	Cat# SAB1412738	#12139-7F8	NA	1:50	Sigma
A1CF	Cat# SAB1400523	#HC271	NA	1:50	Sigma
NEUROD1	Cat# 563566	#8352861	R8-294	1:50	BD Biosciences
NKX2.2	Cat# 564729	#9031625	74.5A5	1:50	BD Biosciences

Secondary Ab

PE Donkey anti-rabbit	Cat# 406421	#B233152	poly4064	1:125	Biolegend
BV421 Goat anti-rat	Cat# 565013	#1085807	NA	1:125	BD Biosciences
APC Goat anti-mouse	Cat# A865	#2384050	NA	1:125	Invitrogen

Cut and Run Ab

HNF1A	Cat# ab204306	#1003158-2	NA	1ug	abcam

Supplementary Table 6. Patient Donor Information used in this study

Islets used in scRNA-seq/scATAC-seq				
Donor	HbA1C	BMI	Age (yrs)	Sex
H1	5.8	31.9	45	M
H2	5.6	24.2	24	M
H3	5.4	30.7	62	F
H4	5.8	35.1	41	M
H5	5.6	28.5	55	M
H6	5.2	23.5	41	M
H7	5.4	23.2	43	F
T2D1	6.7	32.5	58	M
T2D2	5.9	31.7	63	M
T2D3	7.6	24.8	52	M
T2D4	6.7	25.8	53	M

Islets used in Flow Cytometry					
Donor	HbA1C	BMI	Age (yrs)	Sex	Fraction of HNF1A+ β cells
H8	5	31.4	43	F	58.9
H9	4.6	20.8	20	M	75.8
H10	5.4	30.7	62	F	90.4
H7	5.4	23.2	43	F	88.8
T2D1	6.7	32.5	58	M	45.5
T2D5	6.6	31.2	47	M	23.6
T2D3	7.6	24.8	52	M	70.1
T2D4	6.7	25.8	53	M	59.1
