# Supplementary material for: Single cell multiomic analysis reveals diabetes-associated β-cell heterogeneity driven by HNF1A
Source: Nat Commun. 2023 Sep 5;14:5400. doi: 10.1038/s41467-023-41228-3 (PMC10480445; doi:10.1038/s41467-023-41228-3)
Supplement: Supplementary file 3 — Description of Additional Supplementary Files [file 41467_2023_41228_MOESM3_ESM.pdf]

Description of Additional Supplementary Files

Supplementary Data 1: Islet cell-type specific gene expression

Supplementary Data 2: Islet cell-type specific peaks

Supplementary Data 3: Type II Diabetes trajectory genes and peaks identified by RePACT

Supplementary Data 4: Key Type II Diabetes transcription factors and their regulatory networks

Supplementary Data 5: QC for scRNA-seq and snATAC-seq

Supplementary Data 6: Chromatin loops in islet alpha and beta cells
