# Supplementary material for: Single cell multiomic analysis reveals diabetes-associated β-cell heterogeneity driven by HNF1A
Source: Nat Commun. 2023 Sep 5;14:5400. doi: 10.1038/s41467-023-41228-3 (PMC10480445; doi:10.1038/s41467-023-41228-3)
Supplement: Supplementary file 10 — Reporting Summary [file 41467_2023_41228_MOESM10_ESM.pdf]

nature portfolio

Corresponding author(s): Yan Li, Fulai Jin

Last updated by author(s): 08-12-2023

Reporting Summary

Nature Portfolio wishes to improve the reproducibility of the work that we publish. This form provides structure for consistency and transparency in reporting. For further information on Nature Portfolio policies, see our Editorial Policies and the Editorial Policy Checklist.

Statistics

For all statistical analyses, confirm that the following items are present in the figure legend, table legend, main text, or Methods section.

n/a	Confirmed	
☐	☒	The exact sample size (*n*) for each experimental group/condition, given as a discrete number and unit of measurement
☐	☒	A statement on whether measurements were taken from distinct samples or whether the same sample was measured repeatedly
☐	☒	The statistical test(s) used AND whether they are one- or two-sided<br>*Only common tests should be described solely by name; describe more complex techniques in the Methods section.*
☒	☐	A description of all covariates tested
☐	☒	A description of any assumptions or corrections, such as tests of normality and adjustment for multiple comparisons
☐	☒	A full description of the statistical parameters including central tendency (e.g. means) or other basic estimates (e.g. regression coefficient) AND variation (e.g. standard deviation) or associated estimates of uncertainty (e.g. confidence intervals)
☐	☒	For null hypothesis testing, the test statistic (e.g. *F*, *t*, *r*) with confidence intervals, effect sizes, degrees of freedom and *P* value noted<br>*Give P values as exact values whenever suitable.*
☒	☐	For Bayesian analysis, information on the choice of priors and Markov chain Monte Carlo settings
☒	☐	For hierarchical and complex designs, identification of the appropriate level for tests and full reporting of outcomes
☐	☒	Estimates of effect sizes (e.g. Cohen's *d*, Pearson's *r*), indicating how they were calculated

Our web collection on statistics for biologists contains articles on many of the points above.

Software and code

Policy information about availability of computer code

Data collection	No software used.
Data analysis	All software information is also included in the methods.<br>We used: R 3.6.0, STAR v2.5.l, bowtie2 v2.2.6 , Bowtie vl.1.2, picard vl.93, bamtobed vl.2.0, MACS2 v2.2.7.l , Seurat v3 , samtools vl.3.1, bedtools v 2.25 , qvalue, chromVAR, gage, Hocomoco vll.<br>Custom code: Deeploop (https://github.com/JinlabBioinfo/Deeploop), all others (https:// github.com/JinlabBioinfo/RePACT)

For manuscripts utilizing custom algorithms or software that are central to the research but not yet described in published literature, software must be made available to editors and reviewers. We strongly encourage code deposition in a community repository (e.g. GitHub). See the Nature Portfolio guidelines for submitting code & software for further information.

Data

Policy information about availability of data

All manuscripts must include a data availability statement. This statement should provide the following information, where applicable:

- Accession codes, unique identifiers, or web links for publicly available datasets
- A description of any restrictions on data availability
- For clinical datasets or third party data, please ensure that the statement adheres to our policy

The scRNA-seq, snATAC-seq and eHi-C data can be found in accession number GSE195523. The Cut&Run data can be found in accession number GSE234754.

Patchseq
data was obtained from GEO accession GSE124742.

Research involving human participants, their data, or biological material

Policy information about studies with human participants or human data. See also policy information about sex, gender (identity/presentation), and sexual orientation and race, ethnicity and racism.

Reporting on sex and gender	Self-reported information on sex of human islet donors is included in Supplementary Table 6
Reporting on race, ethnicity, or other socially relevant groupings	We did not collect or perform any analysis on race/ethnicity of our donors.
Population characteristics	Characteristics of our human islet donors including HbA1C, BMI, Age, Sex is included in Supplementary Table 6.
Recruitment	Human islets samples were acquired from Prodo Laboratories, and limited by sample availability.
Ethics oversight	Studies are IRB exempt.

Note that full information on the approval of the study protocol must also be provided in the manuscript.

Field-specific reporting

Please select the one below that is the best fit for your research. If you are not sure, read the appropriate sections before making your selection.

[x] Life sciences [ ] Behavioural & social sciences [ ] Ecological, evolutionary & environmental sciences

For a reference copy of the document with all sections, see nature.com/documents/nr-reporting-summary-flat.pdf

Life sciences study design

All studies must disclose on these points even when the disclosure is negative.

Sample size	No sample size calculations were performed. Sample size for snATAC-seq and scRNA-seq (7 non-diabetic, 4 diabetic) were limited by availability and comparable to previously published studies (PMID 30865899). For flow cytomtery, samples were also limited by donor availability. We used 4 non-diabetic, 4 diabetic donors, which we think is comparable to our single-cell sample size.
Data exclusions	No data exclusions
Replication	For snATAC-seq, scRNA-seq, and flow cytometry, each donor is considered a biological replicate. Each snATAC-seq and scRNA-seq experiment was performed independently. For flow cytometry, the 8 samples were performed in three separate batches. For qPCR and GSIS experiments in endoc-bh3 cells, we used at least 3 biological replicates performed on separate days or using separate passage number of cells. Each biological replicate includes at least 3 technical replicates. All attempts at replication were successful.
Randomization	Randomization was not relevant to this study. We assessed differences between healthy and diseased individuals. We did not perform any treatments on these samples that would require randomization.
Blinding	The identity of donors is blinded to the investigators.

Reporting for specific materials, systems and methods

We require information from authors about some types of materials, experimental systems and methods used in many studies. Here, indicate whether each material, system or method listed is relevant to your study. If you are not sure if a list item applies to your research, read the appropriate section before selecting a response.

Materials & experimental systems

n/a	Involved in the study	
[ ]	[x]	Antibodies
[ ]	[x]	Eukaryotic cell lines
[x]	[ ]	Palaeontology and archaeology
[x]	[ ]	Animals and other organisms
[x]	[ ]	Clinical data
[x]	[ ]	Dual use research of concern
[x]	[ ]	Plants

Methods

n/a	Involved in the study	
[ ]	[x]	ChIP-seq
[ ]	[x]	Flow cytometry
[x]	[ ]	MRI-based neuroimaging

Antibodies

Antibodies used	Detailed antibody information for flow cytometry is included in Supplementary Table 6. For flow cytometry: primary antibodies (HNF1A Cat# 896705 lot#1, CST, Cpep Cat# GN-ID4-s, DSHB, TTR Cat# sc-377517 lot#J0316, Santa Cruz, Six3 Cat# sc-398797 lot#B0922, Santa Cruz, TMED6 Cat# SAB1408365, lot#10027 Sigma, PKIB Cat# SAB1412738 lot#12139-7F8, Sigma, A1CF Cat# SAB1400523 lot#HC271, Sigma, NeuroD1 Cat# 563566 lot#8352861 clone#R8-294, BD Biosciences, NKX2.2 Cat# 564729 lot#9031625 Clone#74.5A5, BD Biosciences.) Secondaries (Cat# 406421, Biolegend, Cat# 565013 lot#1085807, BD Biosciences, Cat# A865 lot#2384050, Invitrogen.) HNF1A Cut and Run (Abcam #ab204306, lot#1003158-2)
Validation	HNF1A Cat# 896705, CST: Manufacturer's website states the antibody is specific for human, mouse, rat, validated for IF, WB, and IP<br>Cpep Cat# GN-ID4-s: Manufacturer's website states this antibody is specific for human, monkey and validated in FACS, IF, IHC, IP<br>TTR Cat# sc-377517: Manufacturer's website states this antibody is specific for human, rat, mouse, recommended for WB, IP, IF, IHC(P) and ELISA.<br>Six3 Cat# sc-398797: Manufacturer's website states this antibody is specific for human, rat, mouse, recommended for WB, IP, IF and ELISA.<br>TMED6 Cat# SAB1408365: Manufacturer's website states this antibody is specific for human, recommended for WB.<br>PKIB Cat# SAB1412738: Manufacturer's website states this antibody is specific for human, recommended for WB, ELISA.<br>A1CF Cat# SAB1400523: Manufacturer's website states this antibody is specific for human, recommended for WB, IF.<br>NeuroD1 Cat# 563566: Manufacturer's website states this antibody is specific for human, validated for FACS.<br>NKX2.2 Cat# 564729: Manufacturer's website states this antibody is specific for human, mouse, rat, chicken, validated for FACS, IF.<br>HNF1A #ab204306: Manufacturer's website states this antibody is specific for human, validated for IF, IHC.

Eukaryotic cell lines

Policy information about cell lines and Sex and Gender in Research

Cell line source(s)	EndoC-bh3 cells were obtained from Univercell-Biosolutions
Authentication	No additional authentication was performed
Mycoplasma contamination	All cell lines tested negative for mycoplasma.
Commonly misidentified lines<br>(See ICLAC register)	No commonly misidentified lines used.

Plants

Seed stocks	*Report on the source of all seed stocks or other plant material used. If applicable, state the seed stock centre and catalogue number. If plant specimens were collected from the field, describe the collection location, date and sampling procedures.*
Novel plant genotypes	*Describe the methods by which all novel plant genotypes were produced. This includes those generated by transgenic approaches, gene editing, chemical/radiation-based mutagenesis and hybridization. For transgenic lines, describe the transformation method, the number of independent lines analyzed and the generation upon which experiments were performed. For gene-edited lines, describe the editor used, the endogenous sequence targeted for editing, the targeting guide RNA sequence (if applicable) and how the editor was applied.*
Authentication	*Describe any authentication procedures for each seed stock used or novel genotype generated. Describe any experiments used to assess the effect of a mutation and, where applicable, how potential secondary effects (e.g. second site T-DNA insertions, mosiacism, off-target gene editing) were examined.*

ChIP-seq

Data deposition

- [x] Confirm that both raw and final processed data have been deposited in a public database such as GEO.
- [x] Confirm that you have deposited or provided access to graph files (e.g. BED files) for the called peaks.

Data access links<br>*May remain private before publication.*	https://www.ncbi.nlm.nih.gov/geo/query/acc.cgi?acc=GSE234754
Files in database submission	raw data, processed data (bigwig file)
Genome browser session<br>(e.g. UCSC)	https://genome.ucsc.edu/s/peidong/Plot_Cut_Run_HNF1A_2023_06_06

Methodology

Replicates	NA

Sequencing depth	15M 150bp paired end reads, 2.3M uniquely mapped
Antibodies	HNF1A Cut and Run (Abcam #ab204306)
Peak calling parameters	NA
Data quality	NA
Software	bowtie2 v2.2.6 , samtools v1.3.1, picard v1.93, MACS2 v2.2.7.1 , bedtools v 2.25 (bamtobed,genomecov).

Flow Cytometry

Plots

Confirm that:

- [x] The axis labels state the marker and fluorochrome used (e.g. CD4-FITC).
- [x] The axis scales are clearly visible. Include numbers along axes only for bottom left plot of group (a 'group' is an analysis of identical markers).
- [x] All plots are contour plots with outliers or pseudocolor plots.
- [x] A numerical value for number of cells or percentage (with statistics) is provided.

Methodology

Sample preparation	Human islets were purchased from Prodo Laboratories Inc. Upon receiving, the islets were washed twice with complete PIM (S) Prodo Islet Media (Prodo Laboratories Inc, PIM-S001GMP). Islets were then collected and gently resuspended in complete PIM(S) media and cultured in a 6-well non-tissue culture treated plate overnight. To dissociate islets into single cells, cells were washed once in HBSS (Sigma-aldrich, #6648) and incubated in Accutase (Innovative Cell Technologies, #AT104) at 37°C for 20-25 min. The islets were broken up gently with a 1 ml pipette every 5 min. When >95% of the islets were digested into single cells, PIM(S) medium was added to neutralize the Accutase and the suspension was passed through a 40µm cell strainer. Single cells were washed again with HBSS and fixed and permeabilized with BD cytofix/ cytoperm buffer (BD, Cat# 51-2090KZ) for 20 minutes. Then, cells were stained with selected primary antibodies.
Instrument	BD LSR II, FACS ARIA, or FACS ARIA-SORP
Software	Flowjo v10
Cell population abundance	Typically >80% of total population were cells, >60% of these were single-cells, and ~30% were Cpep+ in non-diabetic donors. In diabetic donors, Cpep+ population can vary from 10-30%
Gating strategy	FSC-A and SSC-H were used to exclude cell debris. Then SSC-A and SSC-H were used to gate out single-cells. Cpep_BV421 and SSC-H was used to select out Cpep+ population. To gate for HNF1A (PE) and target gene (APC), we used minus one controls. For HNF1A (PE) positive, we used APC and Cpep_BV421 staining as negative control. For target gene (APC) positive, we used Cpep_BV421 and HNF1A (PE) staining as negative control.

- [ ] Tick this box to confirm that a figure exemplifying the gating strategy is provided in the Supplementary Information.
